# Evaluation of a newly developed infant chest compression technique: A randomized crossover manikin trial

**DOI:** 10.1097/MD.0000000000005915

**Published:** 2017-04-07

**Authors:** Jacek Smereka, Karol Bielski, Jerzy R. Ladny, Kurt Ruetzler, Lukasz Szarpak

**Affiliations:** a Department of Emergency Medical Service, Wroclaw Medical University; b MEDITRANS The Provincial Emergency Medical Service and Sanitary Transport, Warsaw; c Department of Emergency Medicine and Disaster, Medical University Bialystok, Bialystok, Poland; d Department of General Anesthesiology, Anesthesiology Institute, Cleveland Clinic, Cleveland, OH; e Department of Emergency Medicine, Medical University of Warsaw, Warsaw, Poland.

**Keywords:** cardiopulmonary resuscitation, chest compression, infant

## Abstract

**Background:**

Providing adequate chest compression is essential during infant cardio-pulmonary-resuscitation (CPR) but was reported to be performed poor. The “new 2-thumb technique” (nTTT), which consists in using 2 thumbs directed at the angle of 90° to the chest while closing the fingers of both hands in a fist, was recently introduced. Therefore, the aim of this study was to compare 3 chest compression techniques, namely, the 2-finger-technique (TFT), the 2-thumb-technique (TTHT), and the nTTT in an randomized infant-CPR manikin setting.

**Methods:**

A total of 73 paramedics with at least 1 year of clinical experience performed 3 CPR settings with a chest compression:ventilation ratio of 15:2, according to current guidelines. Chest compression was performed with 1 out of the 3 chest compression techniques in a randomized sequence. Chest compression rate and depth, chest decompression, and adequate ventilation after chest compression served as outcome parameters.

**Results:**

The chest compression depth was 29 (IQR, 28–29) mm in the TFT group, 42 (40–43) mm in the TTHT group, and 40 (39–40) mm in the nTTT group (TFT vs TTHT, *P* < 0.001; TFT vs nTTT, *P* < 0.001; TTHT vs nTTT, *P* < 0.01). The median compression rate with TFT, TTHT, and nTTT varied and amounted to 136 (IQR, 133–144) min^–1^ versus 117 (115–121) min^–1^ versus 111 (109–113) min^–1^. There was a statistically significant difference in the compression rate between TFT and TTHT (*P* < 0.001), TFT and nTTT (*P* < 0.001), as well as TTHT and nTTT (*P* < 0.001). Incorrect decompressions after CC were significantly increased in the TTHT group compared with the TFT (*P* < 0.001) and the nTTT (*P* < 0.001) group.

**Conclusions:**

The nTTT provides adequate chest compression depth and rate and was associated with adequate chest decompression and possibility to adequately ventilate the infant manikin. Further clinical studies are necessary to confirm these initial findings.

## 1 Introduction

Cardiac arrest in infants is a rare, but life-threatening event and is associated with an overall mortality of up to 92%.^[1]^ The majority of the infants suffering from cardiac arrest are below the age of 2 years and have poorer chance to survive compared to older children.^[2,3]^ Early and sufficient cardiopulmonary resuscitation (CPR) is the key point, as insufficient CPR is clearly associated with poorer outcome including higher mortality. Chest compressions are crucial in generating circulation to vital organs and providing adequate cerebral and coronary perfusion. Current infant CPR guidelines by the American Heart Association and the European Resuscitation Council (ERC) recommend that the compression depth should be at least one-third of the anterior-posterior diameter of the chest (approximately 4 cm) and the chest compression rate should be between 100 and 120 min^–1^.^[2,3]^ Chest compression rate, chest compression depth, as well as chest release force and compression duty cycles are the 4 main quality measurements to identify adequate CPR.^[4]^

There are 2 established and generally accepted techniques for external chest compressions during CPR in infants: the “2-finger technique” (TFT) and the “2-thumb encircling hands technique” (TTHT).^[5,6]^ Current guidelines suggest the TFT for lone rescuers, whereas the TTHT technique is recommended for 2 rescuers. The major disadvantage of the TFT is due to potential difficulty in alternating between chest compressions and ventilation during CPR.^[5]^ TTHT generates superior arterial pressures compared with the TFT and is often perceived as the more easy and effective chest compression technique.^[7,8]^ However, CPR in infants is generally poorly performed, even by advanced pediatric life support (APLS) instructors.^[9]^ Therefore, the current CPR strategies, and especially the chest compression techniques must be questioned and there is clearly need for further improvement.

There were some modifications of TTHT for infant CPR, including the vertical 2-thumb technique, which was suggested to generate more pressure than the standard technique in a simulated model of infant out-of-hospital CPR, especially for the rescuers with small hands or a weak grip.^[10]^ Our study group recently published a modified TTHT for infant CPR, the so-called “new 2 thumb technique (nTTT).” The nTTT consists of 2 thumbs directed at the angle of 90° to the chest while closing the fingers of both hands in a fist. Initial data acquired in manikins by physicians are promising, as the nTTT might facilitate adequate chest compression, depth, rate of chest pressure relief, and chest compression rate.^[11]^

Therefore, the aim of this study was to compare the quality of chest compression during infant CPR applied by the nTTT compared to the 2 established chest compression techniques. In particular, we tested the hypothesis that the nTTT is superior compared to the TFT and the TTFT techniques, if performed by skilled paramedics in an infant manikin single rescuer CPR setting.

## 2 Methods

This study was approved by the Institutional Review Board of the Polish Society of Disaster Medicine (approval No.: IRB N18.07.2016). We included 73 paramedics of the Polish Emergency Medical Service (EMS) with at least 1 year of clinical experience in the out-of-hospital emergency setting. All paramedics were actually working in the EMS and are frequently faced with emergency situations, including adult and pediatric CPRs. All paramedics received oral and written information about the scientific and clinical background of this study and were told about the study setting. All paramedics participated voluntarily in this study. Pregnant or paramedics suffering from back pain were not enrolled in this study.

### 2.1 Study design

The study was designed as a randomized crossover manikin trial and was conducted between September and October 2016.

Prior to the study, all the participants received a 30-minute lasting training session on advanced life support (ALS) in infant cardiac arrest according to the current CPR guidelines using standard TFT and TTHT.^[5]^ The nTTT was explained and showed by one of the researchers. A standardized ALS Baby trainer manikin (Laerdal Medical, Stavanger, Norway) simulating a 3-month-old infant was used for all CPR settings. The manikin was placed on a high adjustable hospital stretcher. The bed was leveled to the iliac crest of each rescuer for standardization.

During the evaluation, the participants performed 3 independent CPR settings with the 3 different chest compression techniques (TFT, TTHT, and the nTTT) in an previously randomized sequence.

**Figure 1:**
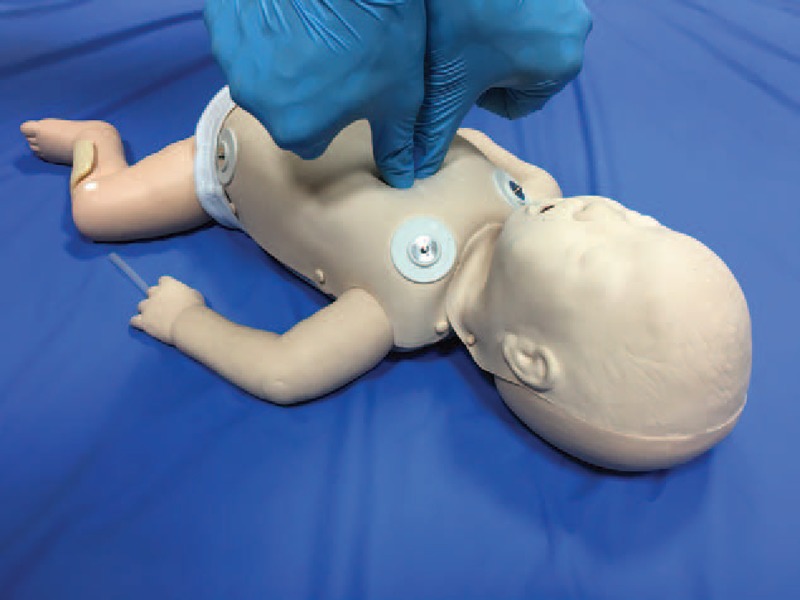
The “new two-thumb technique.”

The 3 chest compression techniques included:

1. The 2 finger technique (TFT). With this method, the rescuer compresses the sternum with the tips of 2 fingers.
2. The 2 thumb technique (TTHT). For this technique, 2 thumbs are placed over the lower third of the sternum, with the fingers encircling the torso and supporting the back.
3. The “new 2-thumb technique” (nTTT). The novel method of CCs in an infant consists in using 2 thumbs directed at the angle of 90° to the chest while closing the fingers of both hands in a fist (Fig. 1).

**Figure 2:**
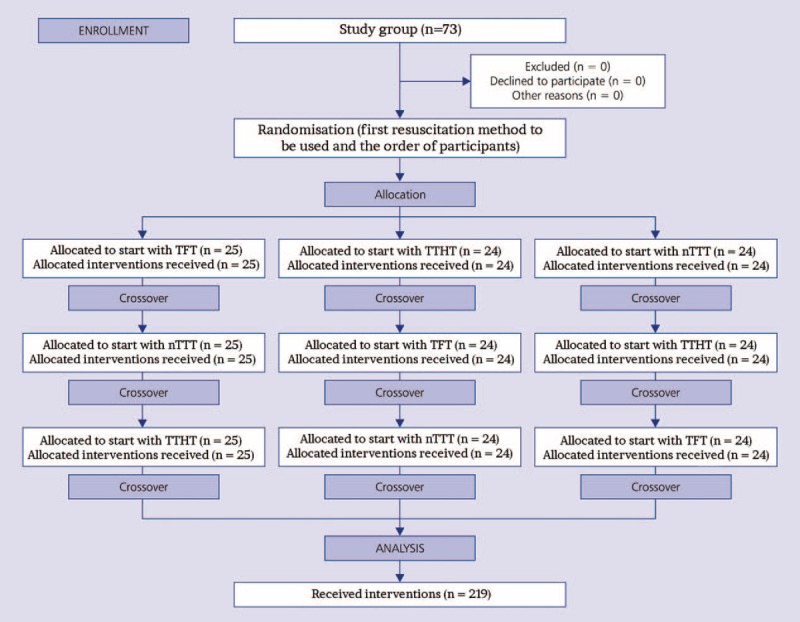
Flow chart of design and recruitment of participants according to CONSORT statement. nTTT = new 2-thumb technique, TFT = 2-finger technique, TTHT = 2-thumb encircling hands technique.

After the training session, the participants were divided into 3 groups with the help of the Research Randomizer software (www.randomizer.org; Fig. 2). Each group was assigned to 1 out of the 3 chest compression techniques. All paramedics performed 2 minutes of CPR according to current infant CPR guidelines including a ratio of 15 chest compression to 2 mouth-to-mouth/nose ventilations. After a break of 20 minutes, paramedics performed the second session with another chest compression technique in the same manner, followed by another 20 minutes break and the last CPR setting.

### 2.2 Data collection

Data on chest compressions were recorded with the Resusci Anne SkillReporter software (Laerdal Medical, Stavanger, Norway). For each resuscitation setting, the total number of chest compressions, mean compression depth, percentage of compression fully released, percentage of compressions deep enough, median rate of all compressions, percentage of compressions with adequate rate, compressions with correct hands position, total ventilations, and median volume were recorded. Additionally, all paramedics were asked about their subjective preference in regard to the chest compression technique they would prefer in the real-life infant CPR setting.

The demographic characteristics of the participants including age, sex, weight, and body mass index were also recorded.

### 2.3 Statistical analysis

Data were analyzed with the use of Statistica software v.12 (StatSoft Inc., Tulsa, OK). The results are shown as numbers (percentages), means and standard deviations (SD), or medians and interquartile ranges (IQR). The occurrence of normal distribution was confirmed by the Kolmogorov–Smirnov test. Analysis of variance (ANOVA) *post hoc* tests with the Bonferroni correction for metric data were used for univariate analysis to compare the 3 study groups. The Kruskal–Wallis test was used to compare non-normally distributed data. Multivariate ANOVA was also applied. The results were considered significant at the level of *P* < 0.05.

## 3 Results

A total of 73 paramedics voluntarily participated in this study. Their median (IQR) age was 30.5 (27–33.5) years, and their average experience in emergency medicine was 4 (2–5.5) years.

**Figure 3:**
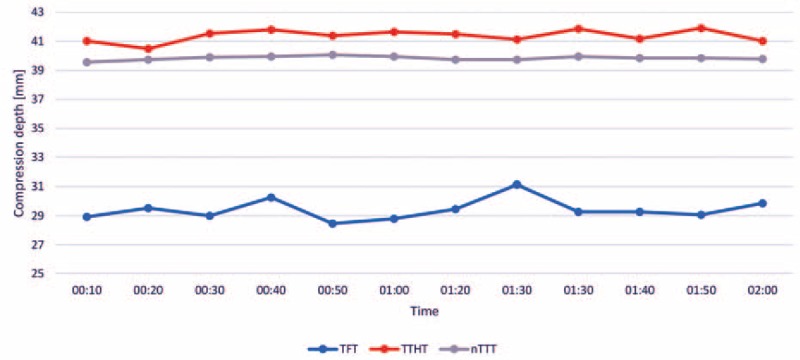
The median chest compression depth using different compression methods.

The chest compression depth equaled 29 (IQR, 28–29) mm with TFT, 42 (40–43) mm with TTHT, and 40 (IQR, 39–40) mm with nTTT (TFT vs TTHT, *P* < 0.001; TFT vs nTTT, *P* < 0.001; TTHT vs nTTT, *P* < 0.01; Table 1; Fig. 3).

**Table 1:**
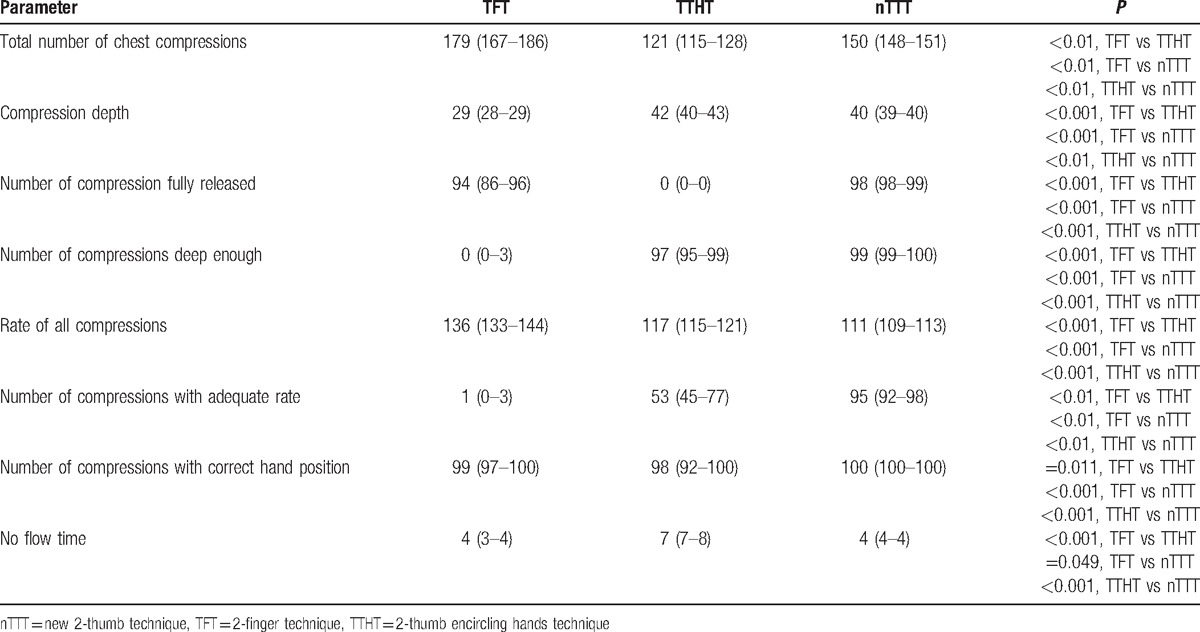
Results of cardiopulmonary resuscitation scenarios.

**Figure 4:**
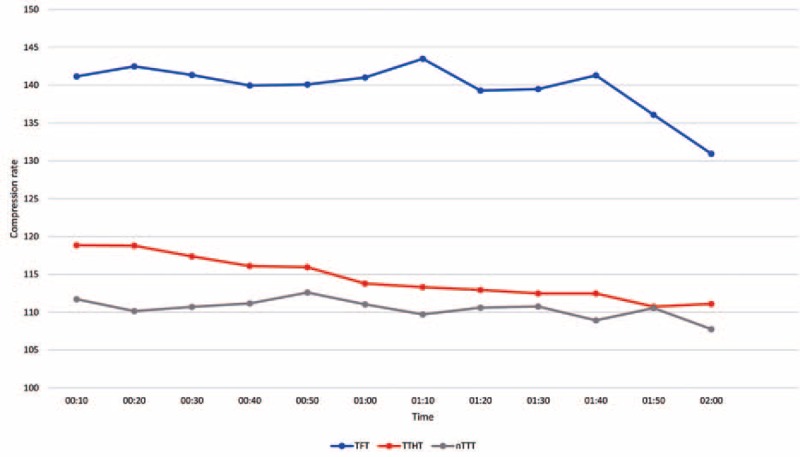
The median chest compression rate using different compression methods.

The median compression rate within the TFT, TTHT, and nTTT varied and amounted to be 136 (IQR, 133–144) min^–1^ versus 117 (115–121) min^–1^ versus 111 (109–113) min^–1^. There was a statistically significant difference in the compression rate between TFT and TTHT (*P* < 0.001), TFT and nTTT (*P* < 0.001), and TTHT and nTTT (*P* < 0.001) (Fig. 4).

Incomplete chest decompressions after chest compressions were significantly increased in the TTHT group compared with the TFT (*P* < 0.001) and the nTTT (*P* < 0.001) group. No flow time was increased with TTHT compared with TFT (*P* < 0.001) and nTTT (*P* < 0.001).

Ventilation parameters in the observed groups varied significantly. Significantly more correct ventilations occurred in the nTTT group compared with TFT and TTHT (*P* < 0.001 and *P* < 0.01, respectively; Table 2). Moreover, the results with nTTT were significantly better than with TFT, as well as with TTHT (*P* < 0.05) for median volume and maximal ventilation volume (Table 2).

**Table 2:**
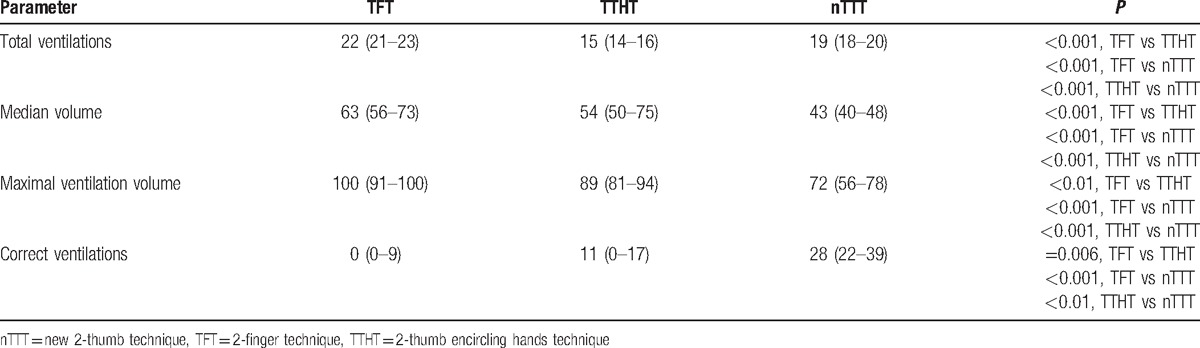
Results of ventilation parameters during cardiopulmonary resuscitation scenarios.

The participants’ subjective assessment of resuscitation with different infant CC techniques showed significant differences between the groups. As for the preferences in hypothetical real life resuscitations, 83% voted for nTTT, 13% for TTHT, and 4% for TFT. There was a statistically significant difference observed between TFT vs TTHT (*P* = 0.006), between TFT vs nTTT (*P* < 0.001), as well as between TTHT vs nTTT (*P* < 0.001).

## 4 Discussion

This study provides novel evidence that the recently introduced nTTT provides adequate chest compression rate and depth in an infant CPR manikin setting. Furthermore, the infant chest was adequate decompressed and subsequent adequate ventilation was enabled. The nTTT was therefore comparable with the established chest compression techniques and even more, somewhat particular superior.

Several studies compared the established 2 chest compression techniques and generally concluded that the TTHT was associated with better results.^[7,8,12,13]^ Many studies revealed the suboptimal chest compression depth and rate with the TFT technique. But there is also increasing evidence that also the TTHT correlates with suboptimal chest compression depth and rate. Several modifications of the chest compression techniques during infant CPR have been proposed to ensure better compliance with current CPR guidelines.^[10]^

High-quality CPR includes 4 “independent” parameters including chest compression rate and depth, chest release force, and compression duty cycles. However, a recent study reported, that even most health care providers do not achieve complete chest recoil during standard CPR.^[14]^ Incomplete chest release during CPR can limit the return of venous blood to the heart, thus reducing coronary and cerebral perfusion pressures. Incomplete chest release is generally observed in infants and children more frequently than in adults.

Very high chest compression rates above 120 min^–1^ is also associated with poorer outcome, but is frequently reported during simulated infant CPR using both TTHT and TFT.^[13]^ These findings are in mild partial contrast with the findings of our study, as the chest compression rate with the nTTT and also the TTHT was well within the recommended threshold (100 to 120 per minute), whereas the TFT completely failed (111 vs 117 vs 136 chest compressions per minute).

High-quality CPR in infants,^[15,16]^ as well as in adults,^[17,18]^ can be a challenge even for certified CPR providers. A constant quality assurance system, including repetitive practical training, is necessary, and the quality of chest compressions is influenced by motor skills and the working environment.^[15]^ It was proved that instantaneous feedback during chest compressions in infants could improve the performance of CC quality during simulated infant CPR.^[15]^

Martin et al^[15]^ evaluated the TTHT and TFT technique in European Pediatric Life Support (EPLS) and/or APLS providers in an simulated, chest-compression-only infant CPR setting, with and without real-time feedback. The compression depth was 42 ± 8 mm in both groups, in the control and in the feedback group using the 2-thumb technique versus 38 ± 6 mm in the control and 38 ± 7 mm in the feedback group using the 2-finger technique, respectively. The chest compression rate were 131 ± 18 min^–1^ in the control and 139 ± 22 min^–1^ in the feedback group using the 2-thumb technique versus 133 ± 24 min^–1^ in the control and 140 ± 20 min^–1^ in the feedback group using the 2-finger technique. Comprehensively, compression depth was acceptable with both techniques, with and without feedback, but chest compression rate was clearly to high and failed to maintain with the recommended thresholds (100 to 120 chest compression per minute). In our study, the mean compression depth was 29 mm in the TFT group versus 42 mm in the TTHT versus 40 mm in the nTTT group, and the mean compression rate equaled was 136 min^–1^ versus 117 min^–1^ versus 111 min^–1^, respectively. The overall performance in regard to chest compression rate and depth was convincing in the nTTT and the TTHT, whereas the TFT failed. Martin et al also reported complete chest decompression after chest compression in 93% to 97% using TFT and 57% to 58% using the TTHT. This finding was not supported by our study, as we found significant worse results in the TTHT compared to the TFT and the nTTT groups.

Another study by Martin et al^[19]^ reported the results of a randomized crossover experimental study comparing the evaluated TTHT and TFT technique in a group of certified APLS instructors performing CPR in infants. The results showed that even in APLS instructors, the mean chest compression rate with both TTHT and TFT exceeded the recommended range (128 ± 21 min^–1^ vs 131 ± 21 min^–1^, respectively). The mean compression depth both with the TTHT and TFT techniques turned out below the recommended level (33 ± 3 mm vs 26 ± 5 mm, respectively). Our study confirms the findings of the TFT group (29 mm). On the other hand, the TTHT (42 mm) was just above the recommended depth of 40 mm, but the nTTT (40 mm) remained within the recommended range.

Udassi et al^[20]^ compared TTHT and TFT during single rescuer infant manikin CPR, reporting a comparable percentage of effective ventilations. The chest compression rate in a 2-minute cycle was 87 min^–1^ in the TTHT group and 92 min^–1^ in TFT. In our study, correct ventilation was achieved mainly in the nTTT group, with low quality in the TFT group and a statistically significantly high total number of CC in TFT. It should be noted that the healthcare providers in the study by Udassi et al^[20]^ were asked to perform 2-minute CPR at the compression rate of 100 min^−1^ applying the compression to ventilation ratio of 30:2.^[20]^

Obtaining chest compression in an adequate rate and depth within the recommended thresholds result in favorable hemodynamic outcomes, such as improved arterial pressures, superior coronary blood flow, and cardiac output.^[19]^ There is also increasing evidence that the overall quality of CPR in infants obtained with both TFT and TTHT is low, regardless of the training level. We believe that our new technique can help to increase the overall CPR quality in infants, allowing a better compliance with international recommendations for infant CPR.

The present study has several limitations. First is the simulation setting using a manikin per se. Even the best manikin and simulation setting cannot entirely simulate real-CPR conditions, especially with regard to the manikin chest compliance, the correlation between the applied force, and the obtained chest compression depth.^[19]^ However, the simulation setting and the manikin we used is accepted as an training instrument and allows us to provide conclusions within the mentioned limitation. Second, we performed the study in a group of experienced paramedics regularly involved in out-of-hospital infant CPR. The results of this study can therefore not transferred without restrictions to all infant-CPR providers. Third, we did not measure any blood pressure and therefore, any conclusion about efficient blood with the nTTT is speculation. However, as the nTTT provides adequate chest compression rate and depth, as well as chest decompression, blood flow should be comparable with the TFT and the TTHT techniques. As a consequence, the results of this study have to be confirmed in an experimental animal setting and several other study groups, including physicians, nurses, and even lay persons with different level of experience in neonatal and infant CPR. Among the strengths of the research, one should mention the comparison of 3 different CPR techniques, including the new nTTT, as well as the randomized crossover nature of the study.

## 5 Conclusions

The nTTT provides adequate chest compression depth and rate and was associated with adequate chest decompression and possibility to adequately ventilate the infant manikin. Further studies in infant CPR including a wide variety of CPR providers experience and clinical settings should investigate this new technique compared to the 2 established chest compression techniques.

## Acknowledgments

The authors would like to thank all paramedic providers for their participation in our study.

## References

[1] Jayaram N McNally B Tang F Survival after out-of-hospital cardiac arrest in children. J Am Heart Assoc 2015;4:e002122. 26450118 10.1161/JAHA.115.002122 PMC4845116

[2] Young KD Gausche-Hill M McClung CD A prospective, population-based study of the epidemiology and outcome of out-of-hospital pediatric cardiopulmonary arrest. Pediatrics 2004;114:157–64. 15231922 10.1542/peds.114.1.157

[3] Kuisma M Suominen P Korpela R Paediatric out-of-hospital cardiac arrests—epidemiology and outcome. Resuscitation 1995;30:141–50. 8560103 10.1016/0300-9572(95)00888-z

[4] Kramer-Johansen J Edelson DP Losert H Uniform reporting of measured quality of cardiopulmonary resuscitation (CPR). Resuscitation 2007;74:406–17. 17391831 10.1016/j.resuscitation.2007.01.024

[5] Maconochie IK Bingham R Eich C European resuscitation council guidelines for resuscitation 2015: Section 6. Paediatric life support. Resuscitation 2015;95:223–48. 26477414 10.1016/j.resuscitation.2015.07.028

[6] de Caen AR Berg MD Chameides L Part 12: Pediatric advanced life support: 2015 American Heart Association Guidelines Update for Cardiopulmonary Resuscitation and Emergency Cardiovascular Care. Circulation 2015;132 (18 suppl 2):S526–42. 26473000 10.1161/CIR.0000000000000266 PMC6191296

[7] Houri PK Frank LR Menegazzi JJ A randomized, controlled trial of two-thumb vs two-finger chest compression in a swine infant model of cardiac arrest [see comment]. Prehosp Emerg Care 1997;1:65–7. 9709339 10.1080/10903129708958789

[8] Dorfsman ML Menegazzi JJ Wadas RJ Two-thumb vs. two-finger chest compression in an infant model of prolonged cardiopulmonary resuscitation. Acad Emerg Med 2000;7:1077–82. 11015237 10.1111/j.1553-2712.2000.tb01255.x

[9] Martin PS Kemp AM Theobald PS Do chest compressions during simulated infant CPR comply with international recommendations? Arch Dis Child 2013;98:576–81. 23193200 10.1136/archdischild-2012-302583

[10] Na JU Choi PC Lee HJ A vertical two-thumb technique is superior to the two-thumb encircling technique for infant cardiopulmonary resuscitation. Acta Paediatr 2015;104:e70–5. 25382371 10.1111/apa.12857

[11] Smereka J Szarpak L Smereka A Evaluation of new two-thumb chest compression technique for infant cardiopulmonary resuscitation performed by novice physicians. A randomized, crossover, manikin trial. Am J Emerg Med 2016;pii: S0735-6757(16)30946-9. doi: 10.1016/j.ajem.2016.12.045. 10.1016/j.ajem.2016.12.045 28040386

[12] David R Closed chest cardiac massage in the newborn infant. Pediatrics 1988;81:552–4. 3353189

[13] Whitelaw CC Slywka B Goldsmith LJ Comparison of a two-finger versus two-thumb method for chest compressions by healthcare providers in an infant mechanical model. Resuscitation 2000;43:213–6. 10711490 10.1016/s0300-9572(99)00145-8

[14] Udassi JP Udassi S Lamb MA Improved chest recoil using an adhesive glove device for active compression-decompression CPR in a pediatric manikin model. Resuscitation 2009;80:1158–63. 19683849 10.1016/j.resuscitation.2009.06.016 PMC4062079

[15] Martin P Theobald P Kemp A Real-time feedback can improve infant manikin cardiopulmonary resuscitation by up to 79%—a randomised controlled trial. Resuscitation 2013;84:1125–30. 23571117 10.1016/j.resuscitation.2013.03.029

[16] Szarpak Ł TZ Smereka J Czyżewski Ł Does the use of a chest compression system in children improve the effectiveness of chest compressions? A randomized crossover simulation pilot study. Kardiol Pol 2016;74:1499–504. 27391911 10.5603/KP.a2016.0107

[17] Zapletal B Greif R Stumpf D Comparing three CPR feedback devices and standard BLS in a single rescuer scenario: a randomised simulation study. Resuscitation 2014;85:560–6. 24215730 10.1016/j.resuscitation.2013.10.028

[18] Kurowski A Szarpak L Bogdanski L Comparison of the effectiveness of cardiopulmonary resuscitation with standard manual chest compressions and the use of TrueCPR and PocketCPR feedback devices. Kardiol Pol 2015;73:924–30. 25985725 10.5603/KP.a2015.0084

[19] Martin PS Kemp AM Theobald PS Does a more “physiological” infant manikin design effect chest compression quality and create a potential for thoracic over-compression during simulated infant CPR? Resuscitation 2013;84:666–71. 23123431 10.1016/j.resuscitation.2012.10.005

[20] Udassi S Udassi JP Lamb MA Two-thumb technique is superior to two-finger technique during lone rescuer infant manikin CPR. Resuscitation 2010;81:712–7. 20227156 10.1016/j.resuscitation.2009.12.029

